# Kinetic Study and Simulation of Titanium Carbide-Supported, Platinum-Doped Tetrahedral Amorphous Carbon Electrodes for Hydrogen Evolution Reaction

**DOI:** 10.3390/ma18091916

**Published:** 2025-04-23

**Authors:** Harunal Rejan Ramji, Nicolas Glandut, Jean-Christophe Orlianges, Joseph Absi, Soh Fong Lim

**Affiliations:** 1 Department of Chemical Engineering and Energy Sustainability, Faculty of Engineering, Universiti Malaysia Sarawak (UNIMAS), Kota Samarahan 94300, Sarawak, Malaysia; rhrejan@unimas.my (H.R.R.); sflim@unimas.my (S.F.L.); 2 Institute for Research on Ceramics (IRCER), UMR 7315, CNRS, University of Limoges, European Ceramics Center, 12 Rue Atlantis, 87068 Limoges, France; 3 XLIM UMR 7252, CNRS, University of Limoges, 123 Avenue Albert THOMAS, 87060 Limoges, France; jean-christophe.orlianges@unilim.fr

**Keywords:** titanium carbide (TiC), tetrahedral amorphous carbon (ta-C), hydrogen evolution reaction (HER), kinetic study, finite element method (FEM), surface diffusion, edge effect

## Abstract

This paper presents the kinetic study of titanium carbide (TiC)-supported, platinum-doped tetrahedral amorphous carbon (taC:Pt) referred to as TiC-taC, for the hydrogen evolution reaction (HER). This study employs the Volmer–Heyrovsky–Tafel (VHT) mechanism. A theoretical approach was utilized to investigate the kinetic properties of these materials for an HER in 0.5 M H_2_SO_4_. TiC-taC exhibited Volmer-dominated reactions with a Tafel slope of 40 mV/dec and the overpotential at 10 mA/cm^2^ was 185 mV. In contrast, isolated TiC and taC:Pt recorded significantly higher Tafel slopes with 60–110 mV/dec and overpotentials of 871 mV and 1009 mV, respectively. The developed model was tested in one dimension (1D) for individual TiC and taC:Pt. The simulated kinetics parameters were determined for both TiC and taC:Pt, revealing that TiC follows the VHT steps, while taC:Pt follows the VH steps. The simulation results show excellent coherence with the experimental results. Further simulation of the hybrid TiC-taC electrocatalyst was conducted considering surface diffusion and edge effects in two (2D) and three dimensions (3D). To the best of our knowledge, this FEM simulation approach is the first to be reported due to the unique geometry of the TiC-taC catalyst enabling the assumption of surface diffusion and edge effect. The introduction of edge effects on the taC:Pt side of the TiC support significantly enhanced the current output, aligning closely with experimental results. The edge exhibited distinct kinetic properties compared to both TiC and taC:Pt. The kinetic parameters determined from the simulation demonstrated strong agreement with experimental findings. Adding the edge effects was essential to explaining the higher current output from the TiC-taC electrode. It exhibited unique kinetic properties not observed in either TiC or taC:Pt alone, acting as a pump where it absorbs *c_Hs_* from neighbouring sites due to surface diffusivity and releases H2 via the Heyrovsky reaction. While surface diffusion had a lesser effect, the simulation indicated its positive influence on the HER.

## 1. Introduction

The hydrogen evolution reaction (HER) stands at the forefront of renewable energy technologies, particularly in the context of hydrogen production for clean energy applications. This electrochemical reaction, which converts water into hydrogen gas, is pivotal for the development of sustainable energy systems. The efficiency of the HER is largely determined by the performance of electrocatalysts [1,2,3], which facilitate the reaction kinetics and overall efficiency of the hydrogen production processes.

Among the various materials explored for HERs, transition metal carbides [4,5,6] and carbon-based materials [7,8,9] have emerged as promising candidates due to their unique electronic properties and catalytic performance. Titanium carbide (TiC), a member of the transition metal carbide family, is known for its hardness, thermal stability, and electrical conductivity, making it a potential candidate for HER applications. A comprehensive study on the electrocatalytic performance of thin-film and bulk TiC conclusively identified TiC as superior to Palladium (Pd) [10], which Shao [11] considered as a good catalyst. In 2021, a study was able to develop a relatively simple method to produce single crystalline TiC with fully exposed {100} crystal planes, demonstrating impressive catalytic activity in HERs [12]. This improved catalytic behaviour is attributed to the partially graphitized carbon shell, which promotes a reduction in the Gibbs free energy for atomic hydrogen adsorption (ΔG), creating a synergy between carbon and TiC. Additionally, TiC as support material has also shown great potential, particularly in HERs. A study found that Pt/TiC exhibited 6.5 times and 3.5 times higher mass activity at a 50 mV overpotential than commercial Pt/C and Pt NP/TiC, respectively [13].

Concurrently, tetrahedral amorphous carbon (ta-C) has garnered attention for its extreme hardness, high electrical conductivity, and chemical stability, which are favourable for catalytic processes [14,15,16]. In many cases, amorphous electrocatalysts have outperformed their crystalline counterparts in electrolysis (Indra et al., 2014) [17]. One reason for this is their flexibility (Liu et al., 2018) [18], which allows them to adapt to electrocatalytic conditions and facilitate both volume and surface-confined electrocatalysis. There is a distinctive difference between amorphous hydrogenated diamond-like carbon with high hydrogen content (a-C:H) and tetrahedral amorphous carbon containing no hydrogen (ta-C)., An extensive study was conducted on the electrochemical properties of undoped ta-C by Laurila et al. (2018) [19] which reveals that the electrical properties of ta-C thin films depend heavily on their thickness and the overall sp2 fraction. The surface of ta-C thin films is consistently sp2-rich despite the underlying material’s sp3 fraction.

Recent advances have demonstrated that atomically dispersed or low-loading Pt-doped electrocatalysts can achieve exceptional hydrogen evolution reaction (HER) activity while minimizing platinum usage. For instance, the deposition of an extremely low concentration of platinum (Pt) into a P-doped Ru alloy catalyst supported on carbon nanotubes (CNTs), denoted as (Ru-P)Pt/C, displayed excellent alkaline hydrogen evolution activity, revealing only 17 mV vs. RHE at a current density of 10 mA/cm^2^ with a Tafel slope value of 27 mV/dec, superior to the commercial Pt/C [20]. The enhanced HER was attributed to the highly diluted Pt atoms dispersed on the surface of Ru nanoparticles through Ru-P-Pt bonds. A study on cobalt–platinum nanoclusters regulated by platinum atomic sites encapsulated in N-doped hollow carbon nanotubes (PtSA-PtCo NCs/N-CNTs) synthesis via pyrolysis exhibits excellent HER catalytic performance, reaching the current density of 10 mA/cm^2^ in 1 M KOH under the low 47 (HER) and 252 mV (OER) overpotentials [21]. Additionally, Pt nanocluster decoration via pulsed laser irradiation in liquid (NC-Pt) is reported to display large surface area, porous structure, high conductivity, abundant active sites, and low Pt content. The electrocatalyst achieves remarkably low HER overpotentials of 52, 57, and 53 mV to attain 10 mA/cm^2^ in alkaline, alkaline seawater, and simulated seawater, surpassing commercial Pt/C catalysts [22]. These advances highlight the potential of Pt-doped systems as a bridge between high-performance and scalable HER electrocatalysts, though challenges remain in precisely controlling dopant coordination and long-term stability under industrial conditions.

In this study, we investigate the HER performance of a novel hybrid catalyst composed of Titanium carbide and platinum-doped tetrahedral amorphous carbon. By combining TiC’s robust catalytic properties [23,24] with the advanced structural and electronic characteristics of ta-C [25], we aim to develop an electrocatalyst with enhanced performance for the HER. This research seeks to explore the kinetic mechanisms underlying the HER on the TiC-ta-C composite, providing insights into the synergistic effects of these materials and paving the way for the design of more efficient electrocatalysts for hydrogen production.

Our approach includes a detailed kinetic study via finite element that examines the reaction rates [26,27,28,29,30] and overall efficiency of the TiC-ta-C composite catalyst under various simulated conditions. Through a combination of electrochemical techniques and structural characterizations, we aim to elucidate the roles of TiC and ta-C in the HER process and identify key factors that influence their catalytic behaviour. At present, the FEM simulation of square arrayed geometries has yet to be presented in any literature.

By bridging the gap between the fundamental understanding of transition metal carbides and amorphous carbon in HER applications [31,32], this research contributes to the broader goal of advancing hydrogen production technologies. Our findings could potentially inform the development of new materials and strategies for more efficient and sustainable hydrogen generation, which is crucial for future clean energy solutions.

## 2. Mathematical Modelling

The fundamental principle of a hydrogen evolution reaction (HER) involves the cathodic electrochemical splitting of water molecules (H_2_O) into hydrogen (H_2_) and oxygen (O_2_), as described by the overall reaction in Equation (1):

(1) $$2H^{+}+2e^{-}\to H_{2}$$

The HER mechanism on electrode surfaces follows the Volmer–Heyrovsky–Tafel (VHT) pathway, represented by Equations (2)–(4)

(2) $$\mathrm{Volmer}:\;H^{+}+s+e^{-}⇄Hs$$

(3) $$\mathrm{Heyrovsky}:\;H^{+}+Hs+e^{-}⇄H_{2}+s$$

(4) $$\mathrm{Tafel}:\;2Hs⇄H_{2}+2s$$

## 3. Volmer–Heyrovsky–Tafel Mechanism

The Volmer and Heyrovsky steps are potential-dependent electrochemical reactions, with rate constants for the forward and backward reactions—e.g., [33] or [34] or [35] expressed as follows:

(5) $$K_{V}=\left(k_{V}e^{\left(-\frac{\beta_{V}nF}{RT}\right)\left(E_{\left(t\right)}-E_{V}^{o}\right)}\right)$$

(6) $$K_{-V}=\left(k_{-V}e^{\left(\frac{\alpha_{V}nF}{RT}\right)\left(E_{\left(t\right)}-E_{V}^{o}\right)}\right)$$

(7) $$K_{H}=\left(k_{H}e^{\left(-\frac{\beta_{H}nF}{RT}\right)\left(E_{\left(t\right)}-E_{H}^{o}\right)}\right)$$

(8) $$K_{-H}=\left(k_{-H}e^{\left(\frac{\alpha_{H}nF}{RT}\right)\left(E_{\left(t\right)}-E_{H}^{o}\right)}\right)$$

where *k_V_* and *k_-V_* are the standard rate constants for forward and backward Volmer steps, respectively, while *K_V_* and *K_-V_* are the rate constants for forward and backward Volmer steps, respectively. The subscript H would represent the Heyrovsky step. The Tafel step is a chemical reaction and not an electrochemical reaction; hence, it is independent of the potential.

Here, *k_V_* and *k_-V_* are the standard rate constants for the Volmer step, and *k_H_* and *k_-H_* are for the Heyrovsky step. The symmetry coefficients are represented by β and α (where α + β = 1). The Tafel step, being a chemical reaction, is independent of the potential.

Using the Langmuir adsorption isotherm, the reaction rates for Equations (2)–(4) are –e.g., [36,37].

(9) $$R_{V}=\left(K_{V}c_{H^{+}}c_{s}\right)-\left(K_{-V}c_{Hs}\right)$$

(10) $$R_{H}=\left(K_{H}c_{H^{+}}c_{Hs}\right)-\left(K_{-H}c_{H_{2}}c_{s}\right)$$

(11) $$R_{T}=\left(K_{T}{\left(c_{Hs}\right)}^{2}\right)-\left(K_{-T}c_{H_{2}}{\left(c_{s}\right)}^{2}\right)$$

Several constants were assumed:

(1) 0.5 M of H_2_SO_4_ of high acidic concentration, hence *c_H+_* = 1000 mol/m^3^.
(2) The maximum surface concentration of the material, $\Gamma_{max}$ = 1 × 10^−5^ mol/m^2^ [38].
(3) The hydrogen concentration near the electrode is 0.001M, c_H2_ = 1 mol/m^3^ [39,40].

COMSOL Multiphysics version 5.5 was used to solve the general partial differential equation (PDE) based on Fick’s second law of diffusion given in Equation (12):

(12) $$e_{a}\frac{\partial^{2}u}{{\partial t}^{2}}+d_{a}\frac{\partial u}{\partial t}=f$$

Setting *e_a_ =* 0 and *d_a_ =* 1 simplifies the PDE for steady-state conditions given by Equation (13):

(13) $$\frac{\partial c_{Hs}}{\partial t}=R_{v}-R_{H}-{2R}_{T}=0$$

Following examples [41,42], the current, *I* (A), is derived from the integrated rate reactions of Volmer and Heyrovsky steps, as expressed in Equations (14) and (15).

(14) $$j=\frac{I_{\sigma }}{A_{\sigma }}=(-nF{\left(R_{V}+R_{H}\right)}_{\sigma })$$

(15) $$I_{\sigma }=-nF\int_{0}^{\sigma_{D}}\int {\left(R_{V}+R_{H}\right)}_{\sigma }d\sigma$$

Hence, the current density *j* (usually measured in mA/cm^2^) is a fundamental parameter to evaluate the electric current flows per unit area of an electrode *A_tot_* in the electrochemical reaction written as Equation (16).

(16) $$j_{Tot}=\frac{I_{Tot}}{A_{Tot}}$$

The Tafel plot can then be obtained by plotting log_10_(*j_Tot_*) against *E(t)*, given in Equation (17).

(17) $$\log_{10}\left(j_{Tot}\right)=\log_{10}\frac{\left(I_{TiC}+I_{taC}\right)}{A_{Tot}}$$

For verification, the analytical equation for an irreversible VH mechanism—e.g., [38] is expressed in Equation (18).

(18) $$J=2F\Gamma_{max}c_{H^{+}}\frac{\left(K_{V}K_{H}\right)}{K_{V}+K_{H}}$$

The robust model was simulated under the numerical parameters provided in Table 1. However, the control parameters can be changed based on different operation conditions or assumptions. For example, in the case of dynamic temperatures, T, electrolyte concentrations, H^+^, and time steps, t.

## 4. Time-Dependent Properties

The overpotential of the system as a function of time *E(t)* is described by Equation (19):

(19) $$E_{\left(t\right)}=\left|v_{b}t+E_{rev}-E_{init}\right|+E_{rev}-E^{o}$$

The overpotential is a measure of additional voltage or energy needed to drive an electrochemical reaction. By setting the scan rate, *v_b_*, at a low value of 1 × 10^−6^ V/s, a slow reaction time simulating a steady-state situation was achieved. This was translated into the software’s transient program with time step, *t_step_* and end time, *t_end_*. The equations used for these parameters are given in Equations (20) and (21).

(20) $$t_{end}=\frac{\left(E_{init}-E_{rev}\right)}{v_{b}}\times 2$$

(21) $$t_{step}=\frac{t_{end}}{no.of\;calculation}$$

The standard potential, E^0^, is specific to the types of electrodes and the mechanistic steps. The simulation is designed to emulate the experimentation of HERs where the cathodic formation of hydrogen occurs at 0 V [39,43].

## 5. Results and Discussion

### 5.1. Finite Element Method (FEM) Simulation

**Figure 1:**
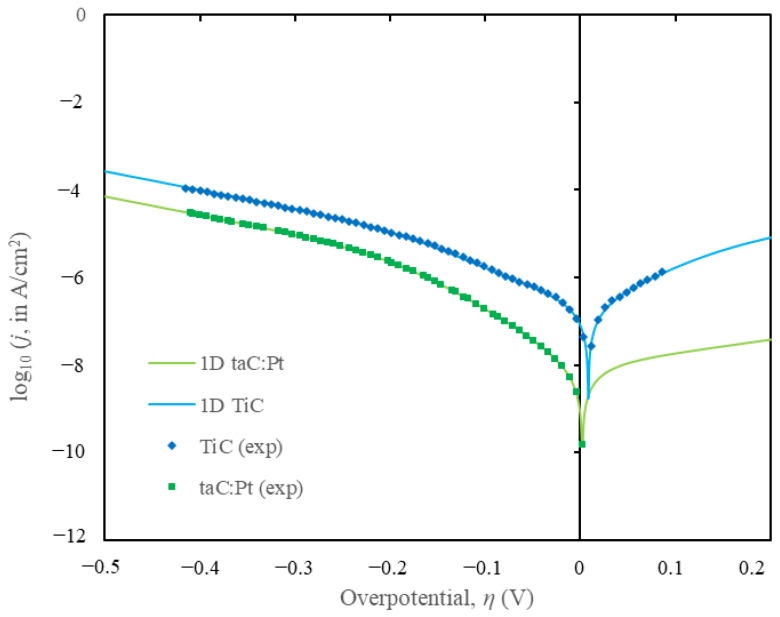
Tafel plots of fitted line from simulation and the experimental curve for log_10_ of current density, (*j* in A/cm^2^) against the cell overpotential, *η* in V.

A finite element method (FEM) model based on the Volmer–Heyrovsky–Tafel (VHT) mechanistic steps was employed to obtain the kinetic parameters for TiC and taC:Pt electrodes. The simulated parameters were compared with experimental Tafel plots [44] for isolated TiC and taC:Pt electrodes. The comparison demonstrated good agreement between the simulated and experimental data, as illustrated in Figure 1.

**Figure 2:**
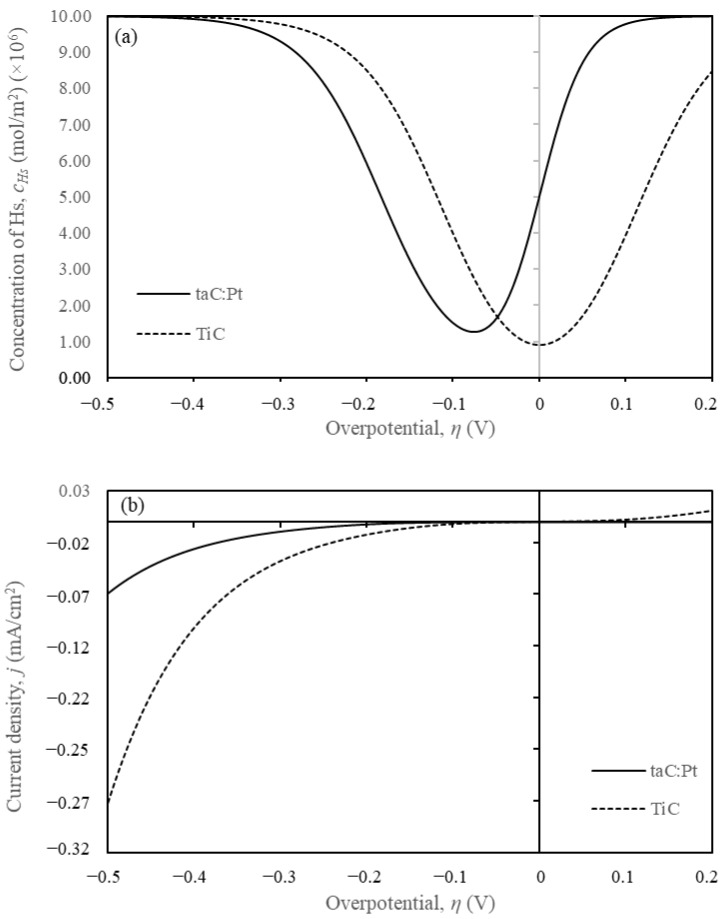
Corresponding (**a**) *c_Hs_* (mol/m^2^) vs. *η* (V) and (**b**) the *j* (mA/cm^2^) vs. *η* (V) for TiC and taC:Pt from Tafel plots Figure 1.

The model assumed reversible reactions and homogeneity across the electrode surface, validating the use of a 1D model. The kinetic parameters derived from the simulations are presented in Table 2. The reverse standard rate constants for the Volmer, Heyrovsky, and Tafel steps (*k_-v_, k_-h_*, and *k_-t_*) were extrapolated from limited experimental data over a potential range of −0.5 V < η < 0.2 V. A minor hump observed at η < 0.05 V on the experimental TiC Tafel plots, attributed to Tafel contrib utions, necessitated adjustments to the reverse kinetic parameters for a better fit. Briefly, the increase of forward kinetics (*k_v_*, *k_h_*, and *k_t_*) will increase the current density, *j* hence shifting the curve to the right (overpotential value closer to zero) in Figure 2b. The inverse response is expected when the forward kinetics are decreased.

In Figure 2a, the c_Hs_ plots reveal that hydrogen is consumed more rapidly on the TiC surface compared to the taC:Pt surface, resulting in higher current densities. At −0.5 V, the current densities are −0.07 mA/cm^2^ for taC and −0.27 mA/cm^2^ for TiC, indicating superior catalytic properties for TiC. The 1D simulation results strongly support the adopted VHT formulation, providing valuable insights into the electrocatalyst properties and catalytic behaviour.

### 5.2. Effect of Surface Diffusion and the Edge Effect Between TiC Substrate and taC

**Figure 3:**
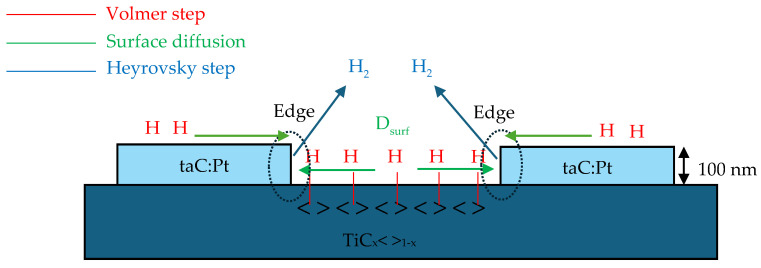
Illustration of VH steps with surface diffusion.

The FEM analysis was employed to study the impact of surface diffusion and edge effect on catalyst performance. Figure 3 illustrates the steps of the Volmer–Heyrovsky–Tafel (VHT) mechanism, incorporating the influence of surface diffusion. Recent research has frequently demonstrated that hybrid or composite materials exhibit superior catalytic performance. Examples by [45,46,47] have provided significant insights into catalytic activity, emphasizing changes in electronic structure and increased active sites from a microscopic perspective. Enhanced catalytic activity has often been attributed to the bonding between the substrate and doped compounds on the electrodes.

**Figure 4:**
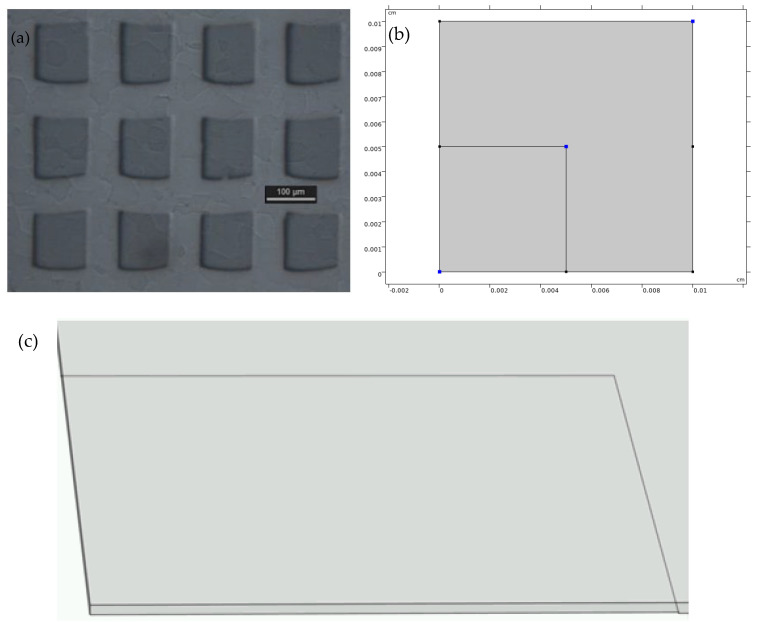
(**a**) Optical micrograph of the TiC/taC:Pt electrode (**b**) 2D model with smaller square on the bottom left-hand corner is the ta-C (**c**) 3D model showing the added depth on the ta-C giving the edge effect.

Figure 4 shows the arrayed structure and distinct surface profiles between TiC and taC:Pt catalysts provide a significant opportunity to geometrically model the electrocatalyst. The macroscopic view of the catalyst was visualized by studying the effect of diffusivity on the surface-adsorbed hydrogen atoms (*c_Hs_*).

The application of surface diffusion alone is insufficient to explain the higher kinetic activity of TiC-taC. To address this, the edge effect was incorporated to account for the observed increase in current from the TiC-taC electrode. This enhancement is supported by—[27,48] studies which indicate higher kinetics at the edges between the two materials. Consequently, the model was revised to include an edge thickness of approximately 100 nm. Equation (22) was utilized to evaluate the current density produced, incorporating the kinetic properties of the edge:

(22) $$j=-nF\left({\left[\left(R_{v}{+R}_{h}\right)A\right]}_{TiC}+{\left[\left(R_{v}{+R}_{h}\right)A\right]}_{taC}+{\left[\left(R_{v}{+R}_{h}\right)A\right]}_{edge}\right)$$

A 3D model was developed to simulate the HER performance, considering both surface diffusion and edge effects. The simulation retained the kinetic parameters for TiC and taC:Pt obtained previously, while the edge kinetic parameters were determined using Equation (22). The surface diffusion effect was modelled under the assumptions that the diffusion coefficients *D_surf_* = *D_TiC_* = *D_taC_* values of 1 × 10^−12^ [49].

This implies that the kinetic properties at the edge are significantly faster than those on the TiC and taC:Pt surfaces (by approximately a factor of 10^4^), overshadowing their contributions. The current output is predominantly influenced by the reaction at the TiC-taC edge.

Finally, the performance of each electrocatalyst was compared: TiC, taC:Pt, and TiC-taC. The experimental curve was well-fitted using the VHT kinetic parameters of all electrocatalysts, as listed in Table 3. The kinetic parameters for the best-performing electrocatalyst, the platinum (Pt) electrode, are also provided for reference, extracted from well-documented studies, e.g., [50].

**Figure 5:**
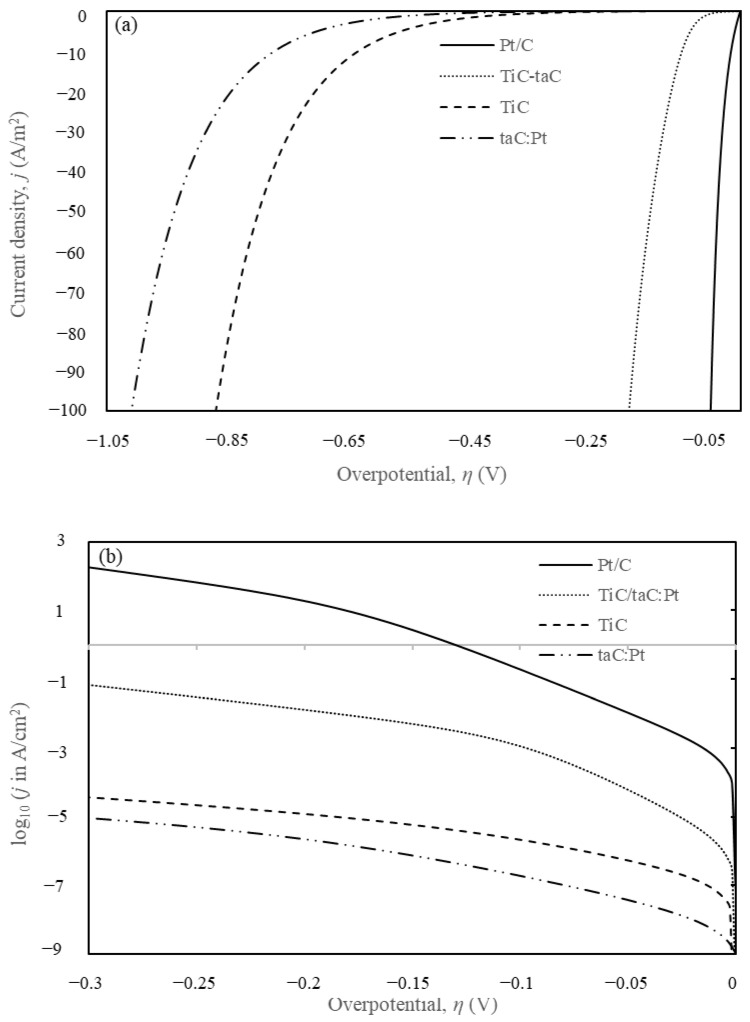
(**a**) HER polarisation curves and (**b**) Tafel plots of TiC, taC:Pt, TiC-taC, and Pt/C.

The current density *j* versus overpotential *η* obtained via simulation was plotted along with the Pt comparison in Figure 5a. The y-axis was capped at 100 A/m^2^ or 10 mA/cm^2^ to provide a clearer view for obtaining η_10_, i.e., η at a current density of 10 mA/cm^2^. The corresponding Tafel plots are shown in Figure 5b. The superior catalytic performance of Pt is evident when compared to the tested materials.

The HER performance of the tested electrocatalysts were recorded in Table 4 along with the comparison of other emerging TiC electroctalysts. The results were evaluated in terms of (1) Tafel slope, (2) overpotentials η at 10 mA/cm^2^, and (3) exchange current density, j_0_ that represents the equilibrium current density where the forward and backward reactions occur at the same rate. Graphically, the j_0_ value can be obtained by extrapolating the linear region of a Tafel plot until the line intercepts η = 0V. TiC-taC and Pt exhibited Volmer-dominated reactions with low Tafel slopes (less than 40 mV/dec) at low η = (10–100 mV) and η_10_ of 185 mV and 50 mV, respectively. In contrast, isolated TiC and taC:Pt recorded significantly higher Tafel slopes (60–110 mV/dec) at low η = (10–200 mV) and η_10_ of 871 mV and 1009 mV, respectively.

**Figure 6:**
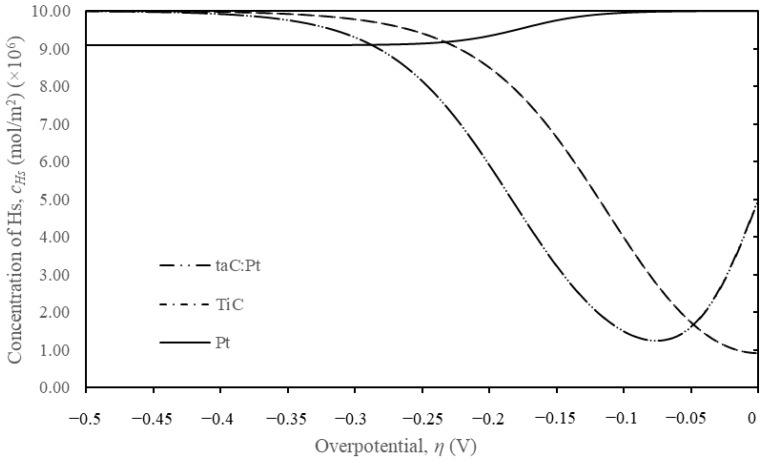
Concentration of adsorbed hydrogen on the electrocatalyst surface.

Through the implementation of edge effects and surface diffusion, the experimental curve of TiC-taC was effectively justified. The developed finite element method (FEM) model successfully estimated the behaviour of the novel TiC-taC catalyst, predicting the concentration of adsorbed hydrogen (c_Hs_) on the catalyst surface, as shown in Figure 6 for taC:Pt, TiC, and Pt modelled on 1D.

**Figure 7:**
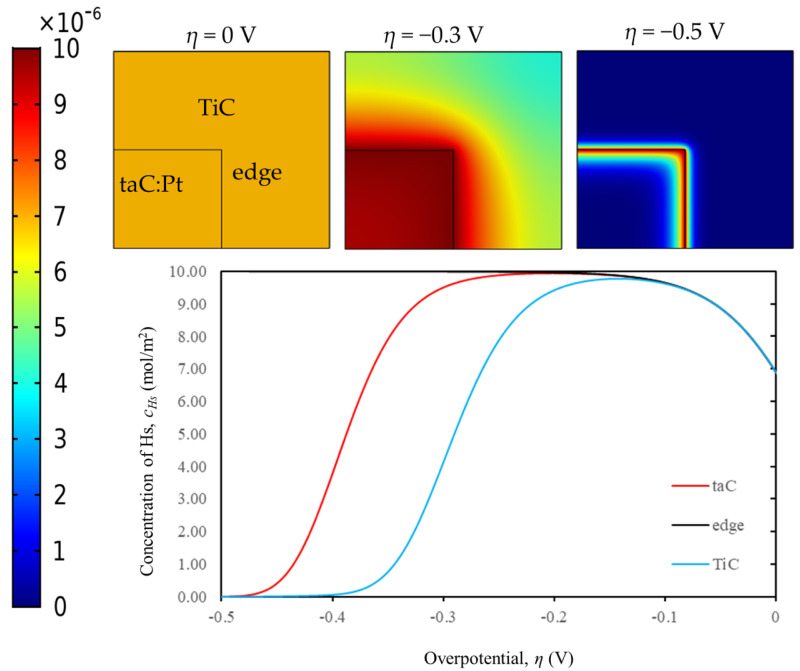
Top view of 3D simulation with surface diffusion and edge effect: The concentration profiles for Hs at *η =* (0, −0.3, −0.5) V and the extracted *c_Hs_* curves at different points of the electrocatalyst surface.

The concentration profile of c_Hs_ is illustrated in Figure 7 for 3D simulations assuming edge effects and surface diffusion. This figure indicates that higher catalytic activity is due to the higher concentration of c_Hs_ at the edges. A decrease in c_Hs_ on TiC and taC:Pt is visible in this region, while c_Hs_ at the edges remained near maximum capacity at Γ_max_ = 1 × 10^−5^ mol/m^2^. Points 1, 5, and 8 in Figure 7 represent taC:Pt (bottom left corner), the edge (the centre point of the square), and TiC (upper right corner), respectively.

**Figure 8:**
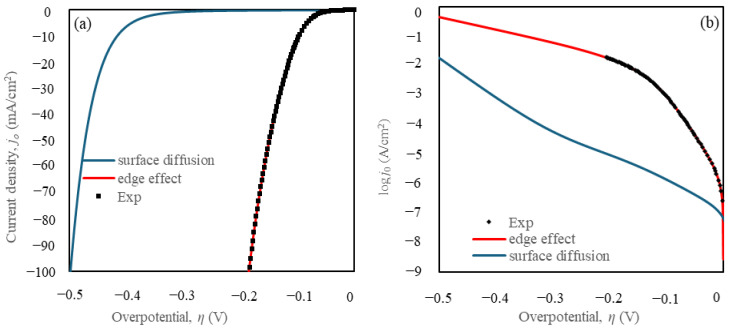
Comparison with the experimental plot on the HER polarization curves for surface diffusion, for edge effect + surface diffusion (**a**) current density, *j* (mA/cm^2^) vs. overpotential, *η* and (**b**) Tafel plots.

The results from the simulation, in terms of the current density *j*_0_, are plotted in Figure 8 in comparison with the experimental curve, using parameters obtained from the simulation provided in Table 3. The simulation plot of the model with edge effects shows excellent coherence with the experimental plot. The edge effect was defined considering the thickness (approximately 100 nm) of the deposited taC:Pt catalyst on the TiC support. This edge does not behave similarly to either TiC or taC:Pt alone. Observations suggest that the edge acts as a pump, absorbing *c_Hs_* from neighbouring sites due to surface diffusivity and releasing H_2_ via the Heyrovsky reaction. This phenomenon is likely due to the enhanced kinetic properties at the edge, resulting from the bonding formation between the catalyst components [50,51,52,53]. Additionally, the possibility of an alloy effect [54] suggests that interactions between Ti and Pt in the catalyst alter the electronic structure and improve charge transfer efficiency.

The resistivity towards corrosion was one of the primary selection criteria for TiC [10] and taC material [55] as electrocatalysts. However, further tests on long-term stability under continuous operation are essential to assess practical viability.

## 6. Conclusions

The developed 1D model accurately determined the kinetic parameters for both TiC and taC, with TiC exhibiting Volmer–Heyrovsky–Tafel (VHT) steps and taC exhibiting Volmer–Heyrovsky (VH) steps. The simulation results demonstrated strong coherence with the experimental findings. For TiC-taC, variations in kinetic parameters with surface diffusivity showed increased current output but did not match experimental results. The inclusion of edge effects was essential to explain the higher current output from the TiC-taC electrode. The edge exhibited unique kinetic properties not observed in either TiC or taC alone, acting as a pump where it absorbs *c_Hs_* from neighbouring sites due to surface diffusivity and releases H_2_ via the Heyrovsky reaction. This study confirmed the reliability and feasibility of using COMSOL to model and verify experimental work, successfully simulating VHT mechanistic steps. The significant impact of edge effects on HER performance suggests the further exploration of different TiC-taC configurations, such as circular taC on TiC support or triangular TiC on taC support. While surface diffusion had a lesser effect, the simulation indicated its positive influence on HERs. This study underlines the importance of electrocatalysts’ surface geometry; further studies on different geometries that maximize the edge effect (for example arrays of circular electrodes with optimized radii and spacing) is recommended.

## Figures and Tables

**Table 1:** Parameters employed for VHT mechanistic steps in the simulation.

Name	Value	Description
*n*	1	No of electron
R	8.314 J/(mol·K)	Universal gas constant
T	298.15 K	Temperature
F	96,485 C/mol	Faraday’s constant
H2	1 mol/m^3^	Hydrogen concentration (c_H2_)
vb	1 × 10^−8^ V/s	Potential scan rate (*v_b_*)
Einit	0.5	Initial potential (*E_init_*)
cstar	1000 mol/m^3^	Initial concentration for H^+^ (*c_H+_*)
Gmax	1 × 10^−5^ mol/m^2^	Maximum concentration of Hs ($\Gamma_{max}$)
tend	2 × 10^8^	Time stop (*t_end_*)
tstep	2 × 10^5^	Time step (*t_step_*)
Erev	−0.5 V	Reverse potential (*E_rev_*)

**Table 2:** Kinetics parameters of TiC electrode and taC electrode.

Electrode	TiC	taC
*k_v_* (m^3^/(mol·s))	6.1 × 10^−7^	4.8 × 10^−8^
*k_-v_* (1/s)	8 × 10^−3^	4.8 × 10^−5^
*k_h_* ((m^3^/(mol·s))	1.09 × 10^−5^	2.8 × 10^−6^
*k_-h_* ((m^3^/(mol·s))	1.09 × 10^−3^	2.8 × 10^−3^
*k_t_* (m^2^/(mol·s))	1 × 10^2^	0
*k_-t_* (m^5^/(mol^2^·s))	1 × 10^3^	0
*β_v_*	0.77	0.82
*β_h_*	0.25	0.25
Area (m^2^)	7.5 × 10^−9^	2.5 × 10^−9^

**Table 3:** Kinetics parameters of TiC electrode and taC:Pt electrode.

Electrode	TiC	taC:Pt	Edge	Pt/C
*k_v_* (m^3^/(mol·s)	6.1 × 10^−6^	4.8 × 10^−8^	9 × 10^1^	3
*k_-v_* (1/s)	8.0 × 10^−2^	4.8 × 10^−5^	9 × 10^1^	3 × 10^7^
*k_h_* (m^3^/(mol·s)	1.09 × 10^−5^	2.8 × 10^−6^	4	3 × 10^1^
*k_-h_* (m^3^/(mol·s)	1.09 × 10^−3^	2.8 × 10^−3^	4 × 10^6^	3
*k_t_* (m^2^/(mol·s)	1 × 10^2^	0	0	0
*k_-t_* (m^5^/(mol^2^·s)	1 × 10^3^	0	0	0
*β_v_*	0.77	0.82	0.9	0.5
*β_h_*	0.25	0.25	0.25	0.5
Area (m^2^)	7.5 × 10^−9^	2.5 × 10^−9^	1 × 10^−11^	

**Table 4:** Summary of the HER performances of the electrocatalyst.

Materials	Tafel Slope (mV/dec)	Overpotentials at 10 mA/cm^2^, η_10_ (mV)	Exchange Current Density, *j_o_* (mA/cm^2^)	Ref.
taC:Pt	60~104 @ −10~−200 mV	−1009	1.26 × 10^−8^	This work
TiC	54~114 @ −10~−200 mV	−871	2.00 × 10^−7^	This work
TiC/taC:Pt	20~40 @ −10~−100 mV	−185	1.00 × 10^−6^	This work
TiC-Ni_SA_	70.3	−149.8	-	[51]
TiC-Co_SA_	69.0	−128.6	-	[51]
TiC-Fe_SA_	61.1	−123.4	-	[51]
Pt/C	15~40 @ −10~−100 mV	−50	2.00 × 10^−3^	[50]

## Data Availability

The original contributions presented in this study are included in the article. Further inquiries can be directed to the corresponding author.
